# Supplementary material for: Rotation to Sparse Loadings Using Lp Losses and Related Inference Problems
Source: Psychometrika. 2023 Mar 31;88(2):527–53. doi: 10.1007/s11336-023-09911-y (PMC10188427; doi:10.1007/s11336-023-09911-y)
Supplement: Supplementary file 1 — (pdf 289 KB) [file 11336_2023_9911_MOESM1_ESM.pdf]

Supplement to "Rotation to Sparse Loadings using L^p Losses and Related Inference Problems"

Symbols

$\mathbf{\Lambda}^*$: $J \times K$ sparse true loading matrix that satisfies Assumption C3.

$\mathbf{\Phi}^*$: $K \times K$ true covariance matrix of the common factors.

$\mathbf{A}^*$: $J \times K$ matrix such that $\mathbf{A}^*\mathbf{A}^{*\prime} = \mathbf{\Lambda}^*\mathbf{\Phi}^*\mathbf{\Lambda}^{*\prime}$.

$\hat{\mathbf{A}}$: Initial estimator of the loading matrix.

$\mathbf{T}$: $K \times K$ rotation matrix.

$\mathcal{M}$: The space of oblique rotation matrices, such that

$$\mathcal{M} = \{\mathbf{T} \in \mathbb{R}^{K\times K} : \mathbf{T}'\mathbf{T} \succ \mathbf{0},\, (\mathbf{T}'\mathbf{T})_{ii} = 1,\, i = 1, \ldots, K\}.$$

Q_p : The family of monotone concave CLFs of the form

$$Q_p(\mathbf{\Lambda}) = \sum_{j=1}^{J}\sum_{k=1}^{K} |\lambda_{jk}|^p.$$

$\hat{\mathbf{T}}$: The solution to the optimisation problem

$$\hat{\mathbf{T}} \in \arg\min_{\mathbf{T}\in\mathcal{M}} Q_p(\hat{\mathbf{A}}\mathbf{T}'^{-1}).$$

g : A bivariate function for a fixed p such that $g : \mathbb{R}^{J\times K} \times \mathcal{M} \to \mathbb{R}$, which maps $g(\mathbf{A}, \mathbf{T}) \to Q_p(\mathbf{A}\mathbf{T}'^{-1})$.

$\mathbf{D}$: $K \times K$ matrix such that the columns of $\mathbf{TD}$ are a permutation of those of $\mathbf{T}$.

$\tilde{\mathbf{D}}$: $K \times K$ matrix such that the k:th column of $\mathbf{T}\tilde{\mathbf{D}}$ is either the same as the k:th column of $\mathbf{T}$ or the k:th column of $\mathbf{T}$ multiplied by -1.

$\mathcal{D}_1$: The set of all $K \times K$ permutation matrices.

$\mathcal{D}_2$: The set of all $K \times K$ sign flip matrices.

$\mathcal{T}^*$: The solution to $\arg\min_{\mathbf{T}\in\mathcal{M}} g(\mathbf{A}^*, \mathbf{T})$. If $\mathbf{T}^* = \mathbf{\Phi}^{*1/2}$, then $\mathbf{T}^*$ is the minimiser of $g(\mathbf{A}^*, \mathbf{T})$, and by Conditions C2 and C3, $\mathcal{T}^* = \{\mathbf{T}^*\mathbf{D}\tilde{\mathbf{D}} : \mathbf{D} \in \mathcal{D}_1, \tilde{\mathbf{D}} \in \mathcal{D}_2\}$.

B_ϵ : $B_\epsilon(\mathbf{T}_0) = \{\mathbf{T} \in \mathcal{M} : ||\mathbf{T}_0 - \mathbf{T}||_2 < \epsilon\}$ denotes the ϵ ball around $\mathbf{T}_0$, and $B_\epsilon(\mathcal{T}^*) = \bigcup_{\mathbf{T}\in\mathcal{T}^*} B_\epsilon(\mathbf{T})$ is the union of the ϵ balls around the elements in $\mathcal{T}^*$.

A Proof of Proposition 1

Proof. On the interval $(0, \infty)$, $h'(x) = px^{p-1} \geqslant 0$ and $h''(x) = p(p-1)x^{p-2} \leqslant 0$ for $p \in (0, 1]$. The function h is hence monotonically increasing and concave on $[0, \infty)$. □

B Proof of Proposition 2

Proof. The inequality in Proposition 2 is already implied by Theorem 1 in Jennrich (2006) combined with Proposition 1. Here, the focus is thus mainly on the equality condition. It is easy to check that if $\mathbf{T}'^{-1} = \mathbf{D}\tilde{\mathbf{D}}$, then $\mathbf{\Lambda}^*\mathbf{T}'^{-1}$ possesses perfect simple structure and $Q_p(\mathbf{\Lambda}^*\mathbf{T}'^{-1}) = Q_p(\mathbf{\Lambda}^*)$. On the other hand, suppose that $\boldsymbol{A} = \mathbf{\Lambda}^*\mathbf{T}'^{-1}$ for some $\mathbf{T} \in \mathcal{M}$ and $Q_p(\boldsymbol{A}) = \min_{\mathbf{T}\in\mathcal{M}} Q_p(\mathbf{\Lambda}^*\mathbf{T}'^{-1}) = Q_p(\mathbf{\Lambda}^*)$. Due to $\mathbf{\Lambda}^* = \boldsymbol{A}\mathbf{T}'$ and since $(\mathbf{T}'\mathbf{T})_{kk} = 1$, $k = 1, \ldots, K$, implies that $||t_k||_2 = 1$ for all columns in $\mathbf{T}$, each row in Λ^* can be expressed as

$$\lambda_j^* = a_{j1}t_1' + a_{j2}t_2' + \ldots + a_{jK}t_K',$$

$j = 1, \ldots, J$. By evaluating the left and right hand side in terms of their ℓ_2 norm, and by applying the triangle inequality, we get that

$$||\lambda_i^*||_2 \leqslant \sum_k |a_{ik}|||t_k'||_2 = \sum_k |a_{ik}|. \tag{B.1}$$

Now, let λ_{is}^* be the only non-zero entry in λ_i^*. By raising it to the p-th power $(0 < p \leqslant 1)$ and applying Lemma 2 in Jennrich (2006),

$$|\lambda_{is}^*|^p \leqslant (\sum_k |a_{ik}|)^p \leqslant \sum_k |a_{ik}|^p. \tag{B.2}$$

Therefore, to achieve $Q_p(\boldsymbol{A}) = Q_p(\mathbf{\Lambda}^*)$, (B.2) needs to hold as an equality for all i, which further implies that (B.1) holds as an equality for all i as well. However, since $t_1, t_2, t_3, \ldots, t_K$ are linearly independent, (B.1) holds as an equality if and only if exactly one of $a_{i1}, a_{i2}, \ldots a_{ik}$ is nonzero for a certain i. Suppose $a_{ij} \neq 0$. Since $\lambda_i^* = a_{ij}t_j'$ and t_j has unit length,

$$t_j = (0, 0, \ldots \frac{\lambda_{is}^*}{a_{ij}}, \ldots, 0)' \in \{+e_s, -e_s\},$$

where e_s is a column vector of length K with 1 on its s:th entry. Since $\text{rank}(\mathbf{T}) = K$, the only possible form of $\mathbf{T}$ is a permutation of $[\pm e_1, \pm e_2, \ldots, \pm e_K]$. Therefore, $\mathbf{T}$ can be written as $\mathbf{D}\tilde{\mathbf{D}}$ for some $\mathbf{D} \in \mathcal{D}_1$ and $\tilde{\mathbf{D}} \in \mathcal{D}_2$. As $\mathbf{T}'^{-1} = \mathbf{D}'^{-1}\tilde{\mathbf{D}}'^{-1}$, we can easily verify that $\mathbf{D}'^{-1} \in \mathcal{D}_1$ and $\tilde{\mathbf{D}}'^{-1} \in \mathcal{D}_2$ and arrive at the result. □

C Proof of Proposition 3

Proof. For any $\gamma > 0$, $\hat{\boldsymbol{\theta}}^{(i)}_{\gamma,p}$ and $\hat{\boldsymbol{\theta}}$ achieve the minimum of $L(\boldsymbol{\Sigma}(\boldsymbol{\theta})) + \gamma Q_p(\boldsymbol{\Lambda})$ and $L(\boldsymbol{\Sigma}(\boldsymbol{\theta}))$ respectively. It follows that

$$L(\boldsymbol{\Sigma}(\hat{\boldsymbol{\theta}})) + \gamma Q_p(\hat{\mathbf{A}}) \geqslant L(\boldsymbol{\Sigma}(\hat{\boldsymbol{\theta}}^{(i)}_{\gamma,p})) + \gamma Q_p(\hat{\boldsymbol{\Lambda}}^{(i)}_{\gamma,p}) \geqslant L(\boldsymbol{\Sigma}(\hat{\boldsymbol{\theta}}^{(i)}_{\gamma,p})) \geqslant L(\boldsymbol{\Sigma}(\hat{\boldsymbol{\theta}})).$$

Therefore, when $\gamma \to 0+$, we have that $L(\boldsymbol{\Sigma}(\hat{\boldsymbol{\theta}})) + \gamma Q_p(\hat{\mathbf{A}}) \to L(\boldsymbol{\Sigma}(\hat{\boldsymbol{\theta}}))$. By the Squeeze theorem (page 104, Sohrab, 2003), $L(\boldsymbol{\Sigma}(\hat{\boldsymbol{\theta}}^{(i)}_{\gamma,p})) \to L(\boldsymbol{\Sigma}(\hat{\boldsymbol{\theta}}))$ when $\gamma \to 0+$. Since $L(\boldsymbol{\Sigma}(\cdot))$ is a continuous function,

$$L(\boldsymbol{\Sigma}(\hat{\boldsymbol{\theta}}^{(i)}_{0,p})) = \lim_{\gamma \to 0+} L(\boldsymbol{\Sigma}(\hat{\boldsymbol{\theta}}^{(i)}_{\gamma,p})) = L(\boldsymbol{\Sigma}(\hat{\boldsymbol{\theta}})) = \min_{\boldsymbol{\theta}} L(\boldsymbol{\Sigma}(\boldsymbol{\theta})).$$

If $\hat{\boldsymbol{\theta}}^{(i)}_{0,p}$ does not solve the optimisation problem in (5) in the main article, there exists a

$$\boldsymbol{\theta}' = (\boldsymbol{\Lambda}', \boldsymbol{\Phi}', \boldsymbol{\Omega}') \text{ s.t. } Q_p(\boldsymbol{\Lambda}') < Q_p(\hat{\boldsymbol{\Lambda}}^{(i)}_{0,p}), \text{ and } L(\boldsymbol{\Sigma}(\boldsymbol{\theta}')) = \min_{\boldsymbol{\theta}} L(\boldsymbol{\Sigma}(\boldsymbol{\theta})).$$

Since Q_p is a continuous function, there exists a γ_0, s.t. $Q_p(\boldsymbol{\Lambda}') < Q_p(\hat{\boldsymbol{\Lambda}}^{(i)}_{\gamma_0,p})$ and $L(\boldsymbol{\Sigma}(\boldsymbol{\theta}')) \leqslant L(\boldsymbol{\Sigma}(\hat{\boldsymbol{\theta}}^{(i)}_{\gamma_0,p}))$, where the latter is because $\boldsymbol{\theta}'$ minimises $L(\boldsymbol{\Sigma}(\boldsymbol{\theta}))$. Therefore,

$$L(\boldsymbol{\Sigma}(\boldsymbol{\theta}')) + \gamma_0 Q_p(\boldsymbol{\Lambda}') < L(\boldsymbol{\Sigma}(\hat{\boldsymbol{\theta}}^{(i)}_{\gamma_0,p})) + \gamma_0 Q_p(\hat{\boldsymbol{\Lambda}}^{(i)}_{\gamma_0,p}),$$

which contradicts that $\hat{\boldsymbol{\theta}}^{(i)}_{\gamma_0,p}$ achieves the minimum of $L(\boldsymbol{\Sigma}(\boldsymbol{\theta})) + \gamma_0 Q_p(\boldsymbol{\Lambda})$. □

D Proof of Theorem 1

We fix p throughout the proof and suppress it as a subscript for all estimators and some of the functions for ease of notation. We will add subscript N when we are considering an estimator applied to a sample of size N. Let $\mathcal{D}(\mathbf{A})$ be the set of all column permutations and sign flips of the matrix $\mathbf{A}$, i.e, $\mathcal{D}(\mathbf{A}) = \{\mathbf{A}\mathbf{D}\tilde{\mathbf{D}} : \mathbf{D} \in \mathcal{D}_1, \tilde{\mathbf{D}} \in \mathcal{D}_2\}$. Let $||\cdot||_{max}$ denote the maximum entry in the matrix, $||\mathbf{A}||_{max} = \max_{i,j} |A_{ij}|$. Let $||\cdot||_2$ denote the matrix norm induced by the vector 2-norm, $||\mathbf{A}||_2 = \max_{||x||_2=1} ||\mathbf{A}x||_2 = \sqrt{\max \operatorname{eig}(\mathbf{A}'\mathbf{A})} = d_1(\mathbf{A})$, where we use $d_k(\mathbf{A})$ to represent the k:th largest singular value of $\mathbf{A}$.

Proof. By Lemma 4, we can find a δ for any $\epsilon_{dist} > 0$, so that as long as $||\mathbf{A} - \mathbf{A}^*||_2 \leqslant \delta$, $\hat{\mathbf{T}} \in B_{\epsilon_{dist}}(\mathcal{T}^*)$. By Lemma 5, there exists a sequence of orthogonal matrices $\{\mathbf{O}_N\}$, such that

$$\hat{\mathbf{A}}_N \mathbf{O}_N \xrightarrow{pr} \mathbf{A}^* \tag{D.1}$$

Therefore, for any $\epsilon_{prob} > 0$, there exists an N_0 so that when $N > N_0$, $\mathbb{P}(||\hat{\mathbf{A}}_N \mathbf{O}_N - \mathbf{A}^*||_2 \leqslant \delta) \geqslant 1 - \epsilon_{prob}$. Consequently, $\mathbb{P}(\hat{\mathbf{T}}_N \in B_{\epsilon_{dist}}(\mathcal{T}^*)) \geqslant 1 - \epsilon_{prob}$, where

$$\hat{\mathbf{T}}_N = \operatorname{argmin}_{\mathbf{T} \in \mathcal{M}} g(\hat{\mathbf{A}}_N \mathbf{O}_N, \mathbf{T}).$$

Thus, by Condition C3, there exists $\mathbf{D}_N \in \mathcal{D}_1$ and $\tilde{\mathbf{D}}_N \in \mathcal{D}_2$ so that $\hat{\mathbf{T}}_N \mathbf{D}_N \tilde{\mathbf{D}}_N \xrightarrow{pr} \mathbf{\Phi}^{*1/2}$. By the continuous mapping theorem, $\hat{\mathbf{T}}_N'^{-1} \mathbf{D}_N'^{-1} \tilde{\mathbf{D}}_N'^{-1} \xrightarrow{pr} \mathbf{\Phi}^{*'-1/2}$. Combined with (D.1) and Slutsky's theorem, we have that

$$\hat{\mathbf{A}}_N (\mathbf{O}_N \hat{\mathbf{T}}_N)'^{-1} \mathbf{D}_N'^{-1} \tilde{\mathbf{D}}_N'^{-1} \xrightarrow{pr} \mathbf{\Lambda}^*,$$

since $\mathbf{O}_N \hat{\mathbf{T}}_N = \text{argmin}_{\mathbf{T} \in \mathcal{M}} g(\hat{\mathbf{A}}_N, \mathbf{T})$ and $\hat{\mathbf{\Lambda}}_N = \hat{\mathbf{A}}_N (\mathbf{O}_N \hat{\mathbf{T}}_N)'^{-1}$. Lastly,

$$\hat{\mathbf{\Phi}}_N = \hat{\mathbf{T}}_N' \hat{\mathbf{T}}_N = \tilde{\mathbf{D}}_N'^{-1} \mathbf{D}_N'^{-1} \mathbf{\Phi}^* \mathbf{D}_N^{-1} \tilde{\mathbf{D}}_N^{-1}$$

where $\mathbf{D}_N'^{-1} \in \mathcal{D}_1, \tilde{\mathbf{D}}_N'^{-1} \in \mathcal{D}_2$, which concludes the proof. □

D.1 Proof of Lemmata 1 to 5

To prove Lemma 4, we will use the property of a continuous function on a compact set. Firstly, let $\mathcal{M}' = \{\mathbf{T} \in \mathbb{R}^{K \times K} : (\mathbf{T}'\mathbf{T})_{kk} = 1, k = 1, \ldots, K\}$. Note that the space of oblique rotation matrices $\mathcal{M}$ can be written as

$$\mathcal{M} = \mathcal{M}' \cap \{\mathbf{T} \in \mathbb{R}^{K \times K} : \text{rank}(\mathbf{T}) = K\}.$$

It follows that $\mathcal{M}$ is not a compact set since $\mathbf{T}$ is invertible, as $d_K(\mathbf{T}) > 0$. In Corollary 1, we therefore first show that if the initial matrix $\hat{\mathbf{A}}$ is in a neighborhood of $\mathbf{A}^*$, i.e, in

$$\bar{\mathcal{B}} = \left\{ \mathbf{A} : ||\mathbf{A} - \mathbf{A}^*||_2 \leqslant \frac{d_K(\mathbf{A}^*)}{2} \right\},$$

then $\hat{\mathbf{T}}$ lies in a compact subset $\overline{\mathcal{M}}$ of $\mathcal{M}$, where

$$\overline{\mathcal{M}} = \mathcal{M}' \cap \left\{ \mathbf{T} \in \mathbb{R}^{K \times K} : d_K(\mathbf{T}) \geqslant \min(\frac{d_K(\mathbf{A}^*)}{4\sqrt{JK} g_{\max}^{1/p}}, 1) \right\}.$$

The maximum $g_{max} = \max_{\mathbf{A} \in \bar{\mathcal{B}}} g(\mathbf{A}, \mathbf{I})$ is attainable since g is continuous and $\bar{\mathcal{B}}$ is compact. Note that $\overline{\mathcal{M}}$ is not empty since $\mathbf{I} \in \overline{\mathcal{M}}$, and $d_K(\mathbf{I}) = 1$.

To prove Corollary 1, we need to prove that if $\mathbf{T}$ is nearly invertible, i.e, its smallest singular value is very small, then it can not be the minimizer of $g(\mathbf{A}, \mathbf{T})$ if $\mathbf{A} \in \bar{\mathcal{B}}$. To make this argument, we will use the matrix inequality in Lemma 1, and Weyl's bound in Lemma 2.

Lemma 1. $||\mathbf{A}\mathbf{T}'^{-1}||_{max} \geqslant \frac{d_K(\mathbf{A})}{\sqrt{JK}} ||\mathbf{T}^{-1}||_2$

Proof. By the norm equivalence of a matrix (chapter 10.4.4, page 62, Petersen & Pedersen, 2012),

$$||\mathbf{A}\mathbf{T}'^{-1}||_{max} \geqslant \frac{1}{\sqrt{JK}} ||\mathbf{A}\mathbf{T}'^{-1}||_2 \tag{D.2}$$

Denote the thin singular value decomposition of $\mathbf{A}$ as $\mathbf{UDV}'$, where $\mathbf{U}$ is a $J \times K$ matrix with orthogonal columns, $\mathbf{D}$ is a $K \times K$ diagonal matrix whose diagonal entries $\mathbf{D}_{kk} = d_i(\mathbf{A})$,

where $d_k(\mathbf{A})$ is the k:th largest singular value of $\mathbf{A}$, and $\mathbf{V}$ is a $K \times K$ orthogonal matrix. When $d_K(\mathbf{A}) = 0$, the statement is trivial, and when $d_K(\mathbf{A}) > 0$, $\mathbf{D}$ is invertible. Therefore

$$\begin{aligned}
||\mathbf{A}\mathbf{T}'^{-1}||_2 &= \sup_{||\mathbf{x}||_2=1} |\mathbf{x}'\mathbf{T}^{-1}\mathbf{V}\mathbf{D}\mathbf{U}'\mathbf{U}\mathbf{D}\mathbf{V}'\mathbf{T}'^{-1}\mathbf{x}|^{1/2} \\
&= ||\mathbf{D}\mathbf{V}'\mathbf{T}'^{-1}||_2 \\
&= \sup_{||\mathbf{x}||_2=1} ||\mathbf{x}'\mathbf{D}||_2 || \frac{\mathbf{x}'\mathbf{D}}{||\mathbf{x}'\mathbf{D}||_2} \mathbf{V}'\mathbf{T}'^{-1}||_2 \\
&\geqslant \inf_{||\mathbf{x}||_2=1} ||\mathbf{x}'\mathbf{D}||_2 \cdot \sup_{||\mathbf{x}||_2=1} || \frac{\mathbf{x}'\mathbf{D}}{||\mathbf{x}'\mathbf{D}||_2} \mathbf{V}'\mathbf{T}'^{-1}||_2 \\
&= d_K \sup_{||\mathbf{y}||_2=1} ||\mathbf{y}'\mathbf{V}'\mathbf{T}'^{-1}||_2 \\
&= d_K ||\mathbf{V}'\mathbf{T}'^{-1}||_2 \\
&= d_K ||\mathbf{T}^{-1}||_2
\end{aligned}$$

Plug $||\mathbf{A}\mathbf{T}'^{-1}||_2 = d_K ||\mathbf{T}^{-1}||_2$ into (D.2) and we get the result. □

Lemma 2 (Weyl's bound, (Weyl, 1912))**.** *For a $J \times K$ matrix $\mathbf{A}$, suppose $\hat{\mathbf{A}} = \mathbf{A} + \mathbf{E}$, where $\mathbf{E}$ represents a perturbation matrix, then we have*

$$\max_{1 \leqslant k \leqslant \min\{J,K\}} |d_k(\mathbf{A}) - d_k(\hat{\mathbf{A}})| \leqslant ||\mathbf{A} - \hat{\mathbf{A}}||_2.$$

We refer interested readers to Theorem 7 in O'Rourke et al. (2018)

Corollary 1. *Under condition C2, $\mathbf{A}^*$ is full rank, so $d_K(\mathbf{A}^*) > 0$. Then, when $\mathbf{A} \in \bar{\mathcal{B}}$,*

$$\arg\min_{\mathbf{T} \in \mathcal{M}} g(\mathbf{A}, \mathbf{T}) \subseteq \overline{\mathcal{M}}$$

Proof. If $\mathbf{T} \in \mathcal{M} \backslash \overline{\mathcal{M}}$, then $||\mathbf{T}^{-1}||_2 = \frac{1}{d_K(\mathbf{T})} > \frac{4\sqrt{JK} g_{\max}^{1/p}}{d_K(\mathbf{A}^*)}$. Thus, by Lemma 1,

$$g(\mathbf{A}, \mathbf{T}) \geqslant (||\mathbf{A}\mathbf{T}'^{-1}||_{max})^p \geqslant (\frac{d_K(\mathbf{A})}{\sqrt{JK}} ||\mathbf{T}'^{-1}||_2)^p > (\frac{4 d_K(\mathbf{A}) g_{\max}^{1/p}}{d_K(\mathbf{A}^*)})^p.$$

When $\mathbf{A} \in \bar{\mathcal{B}}$, by Lemma 2, $|d_K(\mathbf{A}) - d_K(\mathbf{A}^*)| \leqslant ||\mathbf{A} - \mathbf{A}^*||_2 \leqslant \frac{d_K(\mathbf{A}^*)}{2}$, so $d_K(\mathbf{A}) \geqslant \frac{d_K(\mathbf{A}^*)}{2}$. Thus,

$$g(\mathbf{A}, \mathbf{T}) > 2^p g_{max} \geqslant g(\mathbf{A}, \mathbf{I}) \geqslant \min_{\mathbf{T} \in \mathcal{M}} g(\mathbf{A}, \mathbf{T})$$

which contradicts that $\mathbf{T}$ is a minimizer. □

Next, we will prove that if $\mathbf{T}$ is not in a neighborhood of $\mathcal{T}^*$, then there will be a gap between $g(\mathbf{A}^*, \mathbf{T})$ and the minimum.

Lemma 3. *Define $\epsilon_0 = sup\{\epsilon > 0 : \overline{\mathcal{M}} \cap B_\epsilon(\mathcal{T}^*)^{\mathrm{C}} \neq \varnothing\}$, which is achievable since $\overline{\mathcal{M}}$ is compact. Then, for all positive $\epsilon < \epsilon_0$, there exists a $\delta > 0$, such that if $\mathbf{T} \in \overline{\mathcal{M}} \cap B_\epsilon(\mathcal{T}^*)^{\mathrm{C}}$,*

$$g(\mathbf{A}^*, \mathbf{T}) - c^*_{min} \geqslant \delta \qquad \text{(D.3)}$$

where $c^*_{min} \coloneqq \min_{T \in \mathcal{M}} g(\mathbf{A}^*, \mathbf{T})$.

Proof. If the statement does not hold, there exists an $\epsilon' < \epsilon_0$ for all $\delta_m = \frac{1}{m}, m \in \mathbb{N}$, such that $\mathbf{T}_m \in \overline{\mathcal{M}} \cap B_{\epsilon'}(\mathcal{T}^*)^{\mathrm{C}}$. However, $0 < g(\mathbf{A}^*, \mathbf{T}_m) - c^*_{min} < \frac{1}{m}$. Since $\overline{\mathcal{M}} \cap B_{\epsilon'}(\mathcal{T}^*)^{\mathrm{C}}$ is a closed subset of a compact set, it is compact. Therefore, by the Bolzano–Weierstrass theorem (Fitzpatrick, 2009, p.52), there exists a sub-sequence $\{\mathbf{T}_{m_k}\} \subseteq \{\mathbf{T}_m\}$ and a point $\mathbf{T}_0 \in \overline{\mathcal{M}} \cap B_{\epsilon'}(\mathcal{T}^*)^{\mathrm{C}}$, which satisfies $\mathbf{T}_{m_k} \to \mathbf{T}_0$ when $k \to \infty$. However, since $g(\mathbf{A}^*, \mathbf{T})$ is a continuous function of $\mathbf{T}$ when $\mathbf{A}^*$ is fixed, $g(\mathbf{A}^*, \mathbf{T}_0) = \lim_{k\to\infty} g(\mathbf{A}^*, \mathbf{T}_{m_k}) = c^*_{min}$, so $\mathbf{T}_0 \in \mathcal{T}^* \subseteq B_{\epsilon'}(\mathcal{T}^*)$, which makes a contradiction.

□

We can now prove that $\mathbf{T}$ must be close to $\mathcal{T}^*$ if $\mathbf{A}$ is close enough to $\mathbf{A}^*$. We present this result in Lemma 4.

Lemma 4. *Under condition C2, for any $\epsilon < \epsilon_0$, there exists a $\delta > 0$, s.t. if $||\mathbf{A} - \mathbf{A}^*||_2 \leqslant \delta$, then $\mathbf{T} \in B_\epsilon(\mathcal{T}^*)$.*

Proof. For any $\epsilon < \epsilon_0$, let δ_1 be the lower bound of $g(\mathbf{A}^*, \mathbf{T}) - c^*_{min}$ for $\mathbf{T} \in \overline{\mathcal{M}} \cap B_\epsilon(\mathcal{T}^*)^{\mathrm{C}}$ in Lemma 3. Because $\mathbf{\Omega} = \overline{\mathcal{B}} \times \overline{\mathcal{M}}$ is a compact set in the domain of $(\mathbf{A}, \mathbf{T})$ and g is continuous on $\mathbf{\Omega}$, g is uniformly continuous on $\mathbf{\Omega}$. Therefore, for $\frac{\delta_1}{3}$, there exists a $\delta_2 > 0$ s.t. whenever $||\mathbf{A} - \mathbf{A}^*||_2 \leqslant \delta_2$, $|g(\mathbf{A}, \mathbf{T}) - g(\mathbf{A}^*, \mathbf{T})| < \frac{\delta_1}{3}$, for all $\mathbf{T} \in \overline{\mathcal{M}}$. Let $\delta = \min(\frac{d_K(\mathbf{A}^*)}{2}, \delta_2)$. When $||\mathbf{A} - \mathbf{A}^*||_2 \leqslant \delta$ and $\mathbf{T} \in \overline{\mathcal{M}} \cap B_\epsilon(\mathcal{T}^*)^{\mathrm{C}}$,

$$
\begin{aligned}
g(\mathbf{A}, \mathbf{T}) - g(\mathbf{A}, \mathbf{T}^*) \geqslant & (g(\mathbf{A}, \mathbf{T}) - g(\mathbf{A}^*, \mathbf{T})) + (g(\mathbf{A}^*, \mathbf{T}) - g(\mathbf{A}^*, \mathbf{T}^*)) + \\
& (g(\mathbf{A}^*, \mathbf{T}^*) - g(\mathbf{A}, \mathbf{T}^*)) \\
\geqslant & -\frac{\delta_1}{3} + \delta_1 - \frac{\delta_1}{3} = \frac{\delta_1}{3}
\end{aligned}
$$

which means that $\mathbf{T}$ can not be the minimiser. □

In Lemma 5, we prove that after an orthogonal transformation, $\hat{\mathbf{A}}_N$ lies in a small neighborhood of $\mathbf{A}^*$ asymptotically with probability 1.

Lemma 5. *Under conditions C1 and C2, there exists a sequence of orthogonal matrices $\{\mathbf{O}_N\}$ such that $\hat{\mathbf{A}}_N \mathbf{O}_N \xrightarrow{pr} \mathbf{A}^*$.*

Proof. By condition C1, $\hat{\mathbf{A}}_N \hat{\mathbf{A}}'_N \xrightarrow{pr} \mathbf{A}^* \mathbf{A}^{*\prime}$. After multiplying both sides with $\mathbf{A}^*$ and rearranging, we get that

$$\hat{\mathbf{A}}_N \hat{\mathbf{A}}'_N \mathbf{A}^* - \mathbf{A}^* \mathbf{A}^{*\prime} \mathbf{A}^* \xrightarrow{pr} 0.$$

By condition C2, $\mathrm{rank}(\mathbf{A}^{*\prime}\mathbf{A}^*) = \mathrm{rank}(\mathbf{A}^*\mathbf{A}^{*\prime}) = K$, so $(\mathbf{A}^{*\prime}\mathbf{A}^*)^{-1}$ exists. Thus,

$$\hat{\mathbf{A}}_N \hat{\mathbf{A}}'_N \mathbf{A}^* (\mathbf{A}^{*\prime}\mathbf{A}^*)^{-1} - \mathbf{A}^* \xrightarrow{pr} 0. \tag{D.4}$$

Define $\mathbf{B}_N = \hat{\mathbf{A}}'_N \mathbf{A}^* (\mathbf{A}^{*\prime}\mathbf{A}^*)^{-1}$, and $\mathbf{O}_N = \mathbf{B}_N (\mathbf{B}'_N \mathbf{B}_N)^{-1/2}$. Then $\mathbf{O}_N$ is an orthogonal matrix. Therefore, we only need to prove that $\mathbf{O}_N$ forms the desired sequence of matrices. Let $\Delta_N = \mathbf{O}_N - \mathbf{B}_N$. Then

$$
\begin{aligned}
||\hat{\mathbf{A}}_N \mathbf{O}_N - \mathbf{A}^*||_F =& ||\hat{\mathbf{A}}_N (\mathbf{B}_N + \Delta_N) - \mathbf{A}^*||_F \\
\leqslant & ||\hat{\mathbf{A}}_N \mathbf{B}_N - \mathbf{A}^*||_F + ||\hat{\mathbf{A}}_N \Delta_N||_F,
\end{aligned} \tag{D.5}
$$

where $||\cdot||_F$ denotes the Frobenius norm and the first term on the right-hand side of the inequality converges to 0 in probability according to (D.4). For the second term, we have that $||\hat{\mathbf{A}}_N\Delta_N||_F \leqslant ||\hat{\mathbf{A}}_N||_F||\Delta_N||_F$ by the sub-multiplicativity of the Frobenius norm. To control $||\hat{\mathbf{A}}_N||_F$, we can show that under condition C1, $||\hat{\mathbf{A}}_N||_F = \sqrt{tr(\hat{\mathbf{A}}'_N\hat{\mathbf{A}}_N)} = \sqrt{tr(\hat{\mathbf{A}}_N\hat{\mathbf{A}}'_N)} \overset{pr}{\to} \sqrt{tr(\mathbf{A}^*\mathbf{A}^{*\prime})} = ||\mathbf{A}^*||_F$. It is thus bounded. To control Δ_N, we use Theorem 4.1 in Higham (1988), which states that

$$||\Delta_N||_F = \sqrt{\sum_{k=1}^{K}(1-d_i(\mathbf{B}_N))^2} \tag{D.6}$$

where $d_k(\mathbf{B}_N)$ is the k:th largest singular value of $\mathbf{B}_N$. Define $\mathbf{A}^+ = \mathbf{A}^*(\mathbf{A}^{*'}\mathbf{A}^*)^{-1}$ and $\mathbf{E}_N = \hat{\mathbf{A}}_N\hat{\mathbf{A}}'_N - \mathbf{A}^*\mathbf{A}^{*'}$. Then

$$\mathbf{B}'_N\mathbf{B}_N = (\mathbf{A}^{*'}\mathbf{A}^*)^{-1}\mathbf{A}^{*'}(\mathbf{A}^*\mathbf{A}^{*'} + \mathbf{E}_N)\mathbf{A}^*(\mathbf{A}^{*'}\mathbf{A}^*)^{-1} = \mathbf{I} + (\mathbf{A}^{+'})\mathbf{E}_N\mathbf{A}^+, \tag{D.7}$$

and

$$\begin{aligned}\max_{1\leqslant i\leqslant K}|d_i(\mathbf{B}_N)^2 - 1| &= \max_{1\leqslant i\leqslant K}|d_i(\mathbf{B}'_N\mathbf{B}_N) - d_i(\mathbf{I})| \\ &\leqslant ||(\mathbf{A}^+)'\mathbf{E}_N\mathbf{A}^+||_2 \\ &\leqslant ||\mathbf{E}_N||||\mathbf{A}^+||_2^2 \overset{pr}{\to} 0\end{aligned} \tag{D.8}$$

where the first inequality is due to Lemma 2, the second inequality is due to the sub-multiplicativity of the matrix norm, and the convergence is due to $\mathbf{E}_N \overset{pr}{\to} 0$. Therefore, $d_k(\mathbf{B}_N)^2 \overset{pr}{\to} 1$ for $k = 1, 2, .., K$. By the continuous mapping theorem, $d_i(\mathbf{B}_N) \overset{pr}{\to} 1$ for $i = 1, 2, .., K$. Combined with (D.6), we therefore have that $||\Delta_N||_F \overset{pr}{\to} 0$ and $||\hat{\mathbf{A}}_N\mathbf{O}_N - \mathbf{A}^*||_F \overset{pr}{\to} 0$. □

E Proof of Theorem 2

Proof. For a certain threshold $c \in (0, c_0)$, define $\mathbf{\Lambda}^{*(N)} = \mathbf{\Lambda}^*\tilde{\mathbf{D}}_N^{-1}\mathbf{D}_N^{-1} = \{\lambda_{ij}^{*(N)}\}_{J\times K}$ and $E_N = \{||\hat{\mathbf{\Lambda}}_{N,p} - \mathbf{\Lambda}^{*(N)}||_{max} < \min(c, c_0 - c)\}$. By Theorem 1, under conditions C1-C3, $\hat{\mathbf{\Lambda}}_{N,p}\mathbf{D}_N\tilde{\mathbf{D}}_N \overset{pr}{\to} \mathbf{\Lambda}^*$. Therefore, for any $\epsilon > 0$, there exists a N_0 such that when $N > N_0$, $P(E_N) > 1 - \epsilon$. Denote the entries of $\hat{\mathbf{\Gamma}}_{N,p} = \left(\text{sgn}(\hat{\lambda}_{ij}^{(N,p)}) \times 1_{\{|\hat{\lambda}_{ij}^{(N,p)}|>c\}}\right)_{J\times K}$ on E_N as $\hat{\gamma}_{ij}^{(N,p)}$:

$$\hat{\gamma}_{ij}^{(N,p)} = \begin{cases} 0, & \text{if } \lambda_{ij}^{*(N)} = 0, \text{ since } |\hat{\lambda}_{ij}^{N,p}| - 0 < c \\ \text{sgn}(\hat{\lambda}_{ij}^{(N,p)}) = \text{sgn}(\lambda_{ij}^{*(N)}), & \text{if } \lambda_{ij}^{*(N)} \neq 0, \text{ since } |\hat{\lambda}_{ij}^{N,p}| > |\lambda_{ij}^{*(N)}| - (c_0 - c) \geqslant c \end{cases} \tag{E.1}$$

Therefore, when $N > N_0$, $\hat{\mathbf{\Gamma}}_{N,p} = (\text{sgn}(\lambda_{ij}^{*(N)}))_{J\times K} = \mathbf{\Gamma}^*\tilde{\mathbf{D}}_N^{-1}\mathbf{D}_N^{-1}$ with probability at least $1 - \epsilon$. □

F Proof of Theorem 3

Proof. For a fixed s and k, let $A_N = \{\lambda_{sk}^{*(N)} \in (l_{sk}^{(N)}, u_{sk}^{(N)})\}$ be the event of interest, where $\lambda_{sk}^{*(N)}$ are the entries of $\mathbf{\Lambda}^{*(N)} = \mathbf{\Lambda}^*\tilde{\mathbf{D}}_N^{-1}\mathbf{D}_N^{-1}$. Let $B_N = \{\lambda_{sk}^* \in (l_{sk}^{*(N)}, u_{sk}^{*(N)})\}$ be the event of the

confidence interval coverage based on the true sign pattern $\mathbf{\Gamma}^*$. Let $C_N = \{\hat{\mathbf{\Gamma}}_{N,p}\mathbf{D}_N\tilde{\mathbf{D}}_N = \mathbf{\Gamma}^*\}$ be the event that the selected sign pattern is consistent. Since $A_N \cap C_N = B_N \cap C_N$,

$$\mathbb{P}(A_N \cap C_N) = \mathbb{P}(B_N \cap C_N) = \mathbb{P}(B_N) - \mathbb{P}(B_N \cap C_N^C) \xrightarrow{pr} 1 - \alpha,$$

where the limit is due to $P(B_N) \xrightarrow{pr} 1-\alpha$ by condition C6 and $0 \leqslant \mathbb{P}(B_N \cap C_N^C) \leqslant \mathbb{P}(C_N^C) \xrightarrow{pr} 0$ by condition C5. Therefore, combined with $\mathbb{P}(A_N \cap C_N^C) \xrightarrow{pr} 0$ by condition C5,

$$\mathbb{P}(A_N) = \mathbb{P}(A_N \cap C_N) + \mathbb{P}(A_N \cap C_N^C) \xrightarrow{pr} 1 - \alpha.$$

□

G Computational Complexity

For the computational complexity, we remind that we have a loading matrix $\mathbf{\Lambda}$ of dimension $J \times K$, a rotation matrix $\mathbf{T}$ of dimension $K \times K$, a weight matrix $\boldsymbol{W} = \{w_{jk}\}_{J\times K}$ of dimension $J \times K$, and the diagonal of the residual covariance matrix, denoted $\boldsymbol{v}$, which is a vector of dimension $K \times 1$. In order to calculate the computational complexity, we count the number of floating point operations, which includes addition, subtraction, multiplication and division. The following results are simplified by ignoring all terms except the highest order term. We use $O(n)$ to denote a computational complexity of order n, meaning there exists a constant $C > 0$, such that the total number of floating point operations can be controlled by Cn. For example, an $m \times n$ matrix $\mathbf{A}$, $n \times q$ matrix $\mathbf{B}$ and $n \times n$ matrix $\mathbf{C}$, the matrix multiplication operation $\mathbf{AB}$ is of computational complexity $O(mnq)$. By Gauss-Jordan elimination we can also conclude that the inversion of $\mathbf{C}$ is of computational complexity $O(n^3)$.

At iteration t of the proposed IRGP algorithm in Algorithm 3 in the main text, the computations and their complexity are as follows,here we define the approximation function of the objective function of $\boldsymbol{W}$ and $\mathbf{\Lambda}$, by $Q_W(\boldsymbol{W}, \mathbf{\Lambda}) = \sum_{j,k} w_{jk}\lambda_{jk}^2$

$$\begin{aligned}
\mathbf{\Lambda}_t =& \hat{\mathbf{A}}(\mathbf{T}_t')^{-1}, && O(JK^2 + K^3)\\
w_{jk}^{(t)} =& \frac{1}{((\mathbf{\Lambda}_t)_{jk}^2 + \epsilon^2)^{1-p/2}}, && O(JK)\\
\frac{dQ_W(\mathbf{W}, \mathbf{\Lambda}_t)}{d\mathbf{\Lambda}_t} =& 2\mathbf{W} \odot \mathbf{\Lambda}_t, \text{ where } \odot \text{ means element-wise product}, && O(JK)\\
\nabla G_t(\mathbf{T})) =& -(\mathbf{\Lambda}_t' \frac{dQ_W(\mathbf{W}, \mathbf{\Lambda}_t)}{d\mathbf{\Lambda}_t} \mathbf{T}_t^{-1})', && O(JK^2 + K^3)\\
\mathbf{T}_{t+1} =& \text{Proj}(\mathbf{T}_t - \alpha \nabla G_t(\mathbf{T})), && O(K^2)
\end{aligned}$$

Therefore, the per-iteration complexity for Algorithm 3 is $O(JK^2 + K^3)$

At iteration t of the proximal gradient descent algorithm in Algorithm 4 in the main text,

the computations and their complexity is in the following chart

$$\begin{aligned}
\mathbf{\Sigma}(\theta) =& \mathbf{\Lambda}_t\mathbf{T}_t'\mathbf{T}_t\mathbf{\Lambda}' + \text{diag}(\exp(\boldsymbol{v}_t)), && O(J^2K + JK^2 + K^3)\\
\mathbf{Q} =& \mathbf{\Sigma}^{-1} - \mathbf{\Sigma}^{-1}\mathbf{S}\mathbf{\Sigma}^{-1}, && O(J^3)\\
\nabla L_{t,\mathbf{\Lambda}} =& 2\cdot \mathbf{Q}\mathbf{\Lambda}_t\mathbf{T}_t'\mathbf{T}_t, && O(J^2K + JK^2 + K^3)\\
& \text{Prox}_{\alpha,\gamma}(\mathbf{\Lambda}_t - \alpha\nabla L_{t,\mathbf{\Lambda}}), && O(JK)\\
\nabla L_{t,\mathbf{T}_{ij}} =& (2\cdot \mathbf{T}_t\mathbf{\Lambda'}_t\mathbf{Q}\mathbf{\Lambda}_t)_{ij}\mathbf{1}_{\{i\leqslant j\}}, && O(J^2K + JK^2)\\
& \text{Proj}(\mathbf{T}_t - \alpha\nabla L_{t,\mathbf{T}}), && O(K^2)\\
\nabla L_{t,v_{t,i}} =& Q_{ii}\cdot \exp(v_{t,i}), i = 1, ..., J, && O(J)
\end{aligned}$$

Therefore, the per-iteration complexity for Algorithm 4 is $O(J^3 + J^2K + JK^2 + K^3)$.

H Comparison with Other Rotation Criteria

In the following, we demonstrate scenarios where some of the most popular traditional rotation criteria fail to recover the true sparse structure, unlike the proposed criterion. Consider first the Geomin criterion (Yates, 1987), defined as

$$Q_{geo} = \sum_{j=1}^{J}\Big(\prod_{k=1}^{K}\lambda_{jk}\Big)^{\frac{2}{K}}. \tag{H.1}$$

The Geomin criterion thus measures the row-wise complexity and equals zero if at least one entry λ_{jk} in the loading matrix $\mathbf{\Lambda}$, for all $j = 1, \ldots, J$, equals zero. To refrain from indeterminacy of the minimizer, the criterion is commonly modified by adding a small positive constant ϵ, such that

$$Q^{\epsilon}_{geo} = \sum_{j=1}^{J}\Big(\prod_{k=1}^{K}\lambda_{ij}^2 + \epsilon\Big)^{\frac{1}{K}}. \tag{H.2}$$

In the **GPArotation** R package (Bernaards & Jennrich, 2005), (H.2) is the rotation criterion being minimized when the Geomin function is called, with default value $\epsilon = 0.01$.

Consider an initial loading matrix $\boldsymbol{A}$ of dimension 21×3, given in the first three columns of in Table H.1. Notice that the first 15 rows of $\boldsymbol{A}$ contain only one non-zero entry per row, and that the remaining rows contain at least two non-zero entries. Also notice that several of the non-zero entries in the dense part of $\boldsymbol{A}$ are small in magnitude. A majority of the matrix is thus sparse, but with a dense component. One possible solution for the original Geomin criterion in H.1 is given by $\boldsymbol{A}'$, since $Q_{geo}(\boldsymbol{A}') = 0$. This solution is displayed in columns four to six in Table H.1. We verify that $\boldsymbol{A}'$ contains 26 zero entries, whereas $\boldsymbol{A}$ contain 32 zero entries. The dense part of $\boldsymbol{A}$ thus dominates the sparse structure in $\boldsymbol{A}$, making the Geomin criterion unable to recover the true sparse structure. In columns seven to nine in Table H.1, the solution to the adjusted Geomin criterion in (H.2) is presented, with $\epsilon = 0.01$. As displayed, the adjusted Geomin is not either able to recover the true structure of $\boldsymbol{A}$.

We apply the proposed family of rotation criteria, with both $p = 0.5$ and $p = 1$, to the matrix $\boldsymbol{A}$. We verify that the solution is given by $\mathbf{A}$ using grid search over the whole

Table H.1: The initial loading matrix $\boldsymbol{A}$, the transpose of $\boldsymbol{A}$ which is the Geomin solution, and the solution to the adjusted Geomin criterion in (H.2) for a counterexample when the Geomin criterion fails to recover the true sparse structure.

	$\mathbf{A}$			$\mathbf{A}'$			$\arg\min Q_{geo}^{\epsilon=0.01}(\mathbf{A})$		
	F1	F2	F3	F1	F2	F3	F1	F2	F3
1	0.633	0.000	0.000	1.096	0.000	0.895	0.633	0.005	0.014
2	0.000	0.686	0.000	0.000	0.686	0.000	-0.022	0.741	0.317
3	0.000	0.000	0.786	0.000	0.786	1.112	0.014	-0.043	0.769
4	0.954	0.000	0.000	1.653	0.000	1.349	0.954	0.007	0.021
5	0.000	0.601	0.000	0.000	0.601	0.000	-0.019	0.649	0.277
6	0.000	0.000	0.949	0.000	0.949	1.342	0.017	-0.052	0.929
7	0.972	0.000	0.000	1.684	0.000	1.375	0.973	0.008	0.022
8	0.000	0.830	0.000	0.000	0.830	0.000	-0.027	0.897	0.383
9	0.000	0.000	0.815	0.000	0.815	1.152	0.015	-0.045	0.797
10	0.531	0.000	0.000	0.920	0.000	0.751	0.531	0.004	0.012
11	0.000	0.603	0.000	0.000	0.603	0.000	-0.019	0.652	0.278
12	0.000	0.000	0.588	0.000	0.588	0.832	0.011	-0.032	0.575
13	0.844	0.000	0.000	1.461	0.000	1.193	0.844	0.007	0.019
14	0.000	0.692	0.000	0.000	0.692	0.000	-0.022	0.748	0.320
15	0.000	0.000	0.885	0.000	0.885	1.251	0.016	-0.049	0.866
16	0.000	0.117	0.489	0.000	0.606	0.691	0.005	0.100	0.532
17	0.496	-0.165	0.165	0.859	0.000	0.935	0.504	-0.184	0.096
18	0.575	1.138	-0.575	0.996	0.563	0.000	0.528	1.266	-0.024
19	0.000	0.110	0.524	0.000	0.634	0.741	0.006	0.090	0.563
20	0.513	-0.052	0.052	0.889	0.000	0.800	0.516	-0.056	0.039
21	0.559	1.065	-0.559	0.967	0.507	0.000	0.515	1.186	-0.042

oblique rotation matrix space $\mathcal{M}$. When $\boldsymbol{A}$ is used as a starting point for the proposed IRGP algorithm, all of the minimizers of $L^{0.5}$ and L^1 differ at most by a sign flip or column permutation of $\boldsymbol{T} = \mathbf{I}_3$, where $\mathbf{I}_3$ is an identity matrix of dimension 3×3. The true loading matrix $\boldsymbol{A}$ is thus recovered, up to a sign flip and column permutation.

We compare the results with the Quartimin criterion in the Oblimin family and the Promax algorithm as well. The former is defined as

$$Q_{obl} = \sum_{k=1}^{K} \sum_{k' \neq k}^{K} \sum_{j=1}^{J} \lambda_{jk}^2 \lambda_{jk'}^2. \tag{H.3}$$

The oblimin criterion (Harman & Harman, 1976) could thus be understood as a weighted sum related to the complexity of each row of the factor loading matrix. The Promax algorithm (Hendrickson & White, 1964) takes the rotation matrix from Varimax rotation and raises it to powers of 4 in the **stats** R package (Finch, 2006) This has the effect of pushing small values down to zero while larger values are not reduced as much.

In Table H.2, the results of the proposed method for both $p = 0.5$ and $p = 1$, the Geomin and Oblimin criteria, and the Promax algorithm are presented in terms of their MSE. The

starting point for all of the rotation criteria is $\mathbf{A}$. The first column displays the entrywise MSE, calculated as

$$\sum_{ij} \frac{(\mathbf{A}_{ij} - \mathrm{rot}(\mathbf{A})_{ij})^2}{JK},$$

where $\mathrm{rot}(\mathbf{A})$ represents the rotated solution for each respective method. The second column presents the value of the objective function at $\mathbf{A}$, the third column shows the value of rotation criteria at the rotated loading matrix, and the last column the contains the number of zeros produced by the rotated matrix with a cut-off at 0.01. Since Promax is an algorithm that does not involve an objective function, we do not report the objective value for it.

Table H.2: Comparison of the component-wise loss function for $p = 1$ and $p = 0.5$, the Oblimin, the Geomin for $\epsilon = 0.01$ and $\epsilon = 0$, and the Promax rotation methods.

	MSE	Obj	Obj. rot	Number of zeros
L^1	0.000	18.523	18.523	32
$L^{0.5}$	0.000	22.898	22.898	32
Oblimin	0.021	0.896	0.265	2
Geomin($\epsilon = 0.01$)	0.018	1.789	1.354	7
Geomin($\epsilon = 0$)	0.251	1.070	0.000	26
Promax	0.013	-	-	4

As demonstrated in Table H.2, the MSE equals zero for both choices of p for the proposed criterion. The Promax algorithm shows the second to best performance and the Oblimin and Geomin with an $\epsilon = 0.01$ perform similarly. None of the methods, except for the Geomin with $\epsilon = 0$ comes close to the proposed method in terms of identifying the zero elements in the loading matrix, with the proposed method being able to identify all of them for both choices of p.

Lastly, we present the results of the average MSE for each respective rotation method over 500 simulations. The true loading matrix is still $\boldsymbol{A}$ given in Table H.1, and with generated latent factors that are orthogonal to each other. The unique variances of the items corresponds to Item 1-21 in Table I.2 under the column of Item Unique Variance. Three settings are considered, including $N = 400$, 800, and 1600. For each setting, 500 independent simulations are conducted. Table H.3 presents the resulting MSEs, averaged over the number of simulations, and demonstrates the superior performance of the proposed method for both choices of p over the traditional methods.

I True Parameters for Simulation Study I

In this part, the parameters used in Study I are displayed in Table I.1 to Table I.3, including the true loading matrices $\mathbf{\Lambda}^*$, item unique variances $\mathbf{\Omega}^*$ and the lower diagonal part of the true covariance matrices of latent variables $\mathbf{\Phi}^*$ (which are symmetric).

Table H.3: The average MSE for the component-wise loss function for $p = 1$ and $p = 0.5$, the Oblimin, the Geomin for $\epsilon = 0.01$, and the Promax rotation methods, for $N = \{400, 800, 1600\}$.

	$N = 400$	$N = 800$	$N = 1600$
L^1	0.007	0.003	0.002
$L^{0.5}$	0.007	0.003	0.002
Oblimin	0.027	0.024	0.022
Geomin($\epsilon = 0.01$)	0.021	0.019	0.018
Promax	0.018	0.015	0.014

J True Parameters for Study II

The loading matrix $\mathbf{\Lambda}^*$ is shown in Table J.1. The covariance matrix for latent variable is the same as the 15×3 setting in Study I, listed in the last three columns of Table I.3.

K Additional Results for the Big-Five Personality Test Application

Tables K.1 through K.3 show the estimated loading parameters and the corresponding 95% confidence intervals obtained from the L^1 rotation.

Table I.1: 15×3 factor loading patterns $\mathbf{\Lambda}^*$ and item unique variances $\mathbf{\Omega}^*$ in Simulation Study I

	Loading Matrix			Item Unique Variances
	Item 1-15			Item 1-15
	F1	F2	F3	
1	0.71	0	0	1.27
2	0	0.75	0	1.38
3	0	0	0.83	1.57
4	0.96	0	0	1.92
5	0	0.68	0	1.20
6	0	0	0.96	1.90
7	0.98	0	0	1.95
8	0	0.86	0	1.67
9	0	0	0.85	1.63
10	0.62	0.35	0	1.06
11	0	0.68	0.42	1.21
12	0.5	0	0.67	1.17
13	0.87	0	0.31	1.68
14	0.43	0.75	0	1.39
15	0	0.48	0.91	1.77

Table I.2: 30×5 factor loading patterns $\mathbf{\Lambda}^*$ and item unique variances $\mathbf{\Omega}^*$ in Simulation Study I

	Loading Matrix											Item Unique Variances	
	Item 1-15						Item 16-30					Item 1-15	Item 16-30
	F1	F2	F3	F4	F5		F1	F2	F3	F4	F5		
1	0.71	0	0	0	0	16	0.8	0.34	0	0	0	1.27	1.49
2	0	0.75	0	0	0	17	0	0.89	0.38	0	0	1.38	1.72
3	0	0	0.83	0	0	18	0	0	1	0.35	0	1.57	1.99
4	0	0	0	0.96	0	19	0	0	0	0.75	0.26	1.92	1.38
5	0	0	0	0	0.68	20	0.45	0	0	0	0.91	1.20	1.79
6	0.96	0	0	0	0	21	0.97	0	0.4	0	0	1.90	1.93
7	0	0.98	0	0	0	22	0	0.68	0	0.44	0	1.95	1.21
8	0	0	0.86	0	0	23	0	0	0.86	0	0.23	1.67	1.65
9	0	0	0	0.85	0	24	0.42	0	0	0.65	0	1.63	1.13
10	0	0	0	0	0.62	25	0	0.32	0	0	0.71	1.06	1.27
11	0.68	0	0	0	0	26	0.75	0.45	0.39	0	0	1.21	1.39
12	0	0.67	0	0	0	27	0	0.61	0.43	0.37	0	1.17	1.01
13	0	0	0.87	0	0	28	0	0	0.75	0.36	0.44	1.68	1.38
14	0	0	0	0.75	0	29	0.34	0	0	0.95	0.21	1.39	1.88
15	0	0	0	0	0.91	30	0.42	0.41	0	0	0.74	1.77	1.34

Table I.3: The true covariance matrices for latent variables in Simulation Study I.

	30 × 5 setting					15 × 3 setting		
	F1	F2	F3	F4	F5	F1	F2	F3
F1	1					1		
F2	0.085	1				0.021	1	
F3	0.429	0.042	1			0.502	0.274	1
F4	0.148	0.149	0.496	1				
F5	0.249	0.309	0.121	0.19	1			

Table J.1: 18 × 3 true loading matrix and item unique variances in Simulation Study II

	Loading Matrix							Item Unique Variances	
	Item 1-9				Item 10-18			Item 1-9	Item 10-18
	F1	F2	F3		F1	F2	F3		
1	0.531	0.760	0	10	0.124	0.765	0	1.27	1.06
2	0.744	0	0.216	11	0.412	0	0.047	1.38	1.21
3	0	1.870	1.447	12	0	0.681	0.954	1.57	1.17
4	1.816	0.424	0	13	1.374	0.964	0	1.92	1.68
5	0.403	0	1.642	14	0.768	0	1.385	1.20	1.39
6	0	0.251	1.294	15	0	0.987	0.955	1.90	1.77
7	1.889	0.534	0	16	0.995	0.372	0	1.95	1.49
8	1.322	0	1.106	17	1.435	0	0.876	1.67	1.72
9	0	0.027	1.059	18	0	1.337	0.490	1.63	1.99

Table K.1: Part I: Point estimates and confidence intervals constructed by L^1, big-five personality test. The loadings that are significantly different from zero according to the 95% confidence intervals are indicated by asterisks.

	E	ES	A	C	I
E1(+)	0.878*	-0.065	-0.069*	-0.005	0.082*
	(0.793, 0.983)	(-0.158, 0.011)	(-0.180,-0.014)	(-0.134, 0.038)	(0.005, 0.171)
E2(-)	-0.852*	0.127*	0.004	0.048	-0.014
	(-0.975,-0.770)	(0.047, 0.232)	(-0.056, 0.126)	(-0.028, 0.163)	(-0.103, 0.082)
E3(+)	0.785*	0.278*	0.202*	0.099	-0.095*
	(0.692, 0.868)	(0.197, 0.356)	(0.118, 0.274)	(-0.013, 0.148)	(-0.173,-0.018)
E4(-)	-0.922*	-0.063	-0.020	0.022	0.091*
	(-1.026,-0.847)	(-0.144, 0.011)	(-0.072, 0.079)	(-0.030, 0.128)	(0.034, 0.187)
E5(+)	0.889*	-0.024	0.153*	0.083	0.080*
	(0.810, 0.988)	(-0.117, 0.038)	(0.061, 0.212)	(-0.026, 0.131)	(0.022, 0.173)
E6(-)	-0.736*	-0.003	-0.087	-0.043	-0.137*
	(-0.854,-0.661)	(-0.065, 0.113)	(-0.160, 0.012)	(-0.132, 0.051)	(-0.225,-0.050)
E7(+)	1.125*	-0.077*	0.081*	0.081	-0.023
	(1.025, 1.229)	(-0.189,-0.014)	(0.003, 0.174)	(-0.032, 0.143)	(-0.119, 0.052)
E8(-)	-0.710*	-0.095*	0.029	0.138*	-0.064
	(-0.824,-0.628)	(-0.184,-0.002)	(-0.035, 0.143)	(0.059, 0.246)	(-0.156, 0.025)
E9(+)	0.827*	0.057	-0.002	-0.037	0.243*
	(0.737, 0.945)	(-0.045, 0.146)	(-0.108, 0.078)	(-0.174, 0.022)	(0.146, 0.334)
E10(-)	-0.826*	-0.119*	-0.047	-0.099	0.006
	(-0.930,-0.739)	(-0.192,-0.020)	(-0.121, 0.049)	(-0.169, 0.009)	(-0.078, 0.093)
ES1(-)	-0.085*	-0.988*	0.008	0.110*	-0.104*
	(-0.187,-0.003)	(-1.100,-0.895)	(-0.117, 0.079)	(0.067, 0.260)	(-0.195,-0.019)
ES2(+)	0.113*	0.684*	-0.000	-0.106*	0.085*
	(0.001, 0.178)	(0.614, 0.804)	(-0.065, 0.112)	(-0.259,-0.074)	(0.020, 0.194)
ES3(-)	-0.164*	-0.796*	0.233*	0.146*	0.044
	(-0.232,-0.056)	(-0.919,-0.726)	(0.131, 0.308)	(0.109, 0.296)	(-0.039, 0.135)
ES4(+)	0.206*	0.571*	0.000	0.046	0.010
	(0.089, 0.286)	(0.486, 0.688)	(-0.075, 0.118)	(-0.112, 0.090)	(-0.077, 0.116)
ES5(-)	0.056	-0.475*	-0.040	-0.096	-0.228*
	(-0.046, 0.167)	(-0.577,-0.361)	(-0.159, 0.049)	(-0.187, 0.032)	(-0.349,-0.137)
ES6(-)	-0.087	-0.817*	0.259*	-0.001	-0.133*
	(-0.172, 0.007)	(-0.930,-0.736)	(0.154, 0.334)	(-0.030, 0.159)	(-0.231,-0.056)
ES7(-)	0.052	-0.973*	-0.110*	-0.020	0.004
	(-0.008, 0.157)	(-1.077,-0.887)	(-0.213,-0.041)	(-0.059, 0.112)	(-0.090, 0.072)
ES8(-)	0.036	-1.142*	-0.133*	-0.047	0.001
	(-0.021, 0.154)	(-1.259,-1.055)	(-0.247,-0.066)	(-0.076, 0.104)	(-0.104, 0.065)
ES9(-)	0.001	-0.879*	-0.292*	0.195*	-0.016
	(-0.086, 0.092)	(-0.990,-0.795)	(-0.388,-0.207)	(0.145, 0.329)	(-0.110, 0.066)
ES10(-)	-0.332*	-0.846*	0.071	-0.081	0.100*
	(-0.399,-0.220)	(-0.957,-0.765)	(-0.019, 0.163)	(-0.116, 0.068)	(0.011, 0.184)

Table K.2: Part II: Point estimates and confidence intervals constructed by L^1, big-five personality test.

	E	ES	A	C	I
A1(-)	0.002	-0.128*	-0.779*	0.035	0.046
	(-0.118, 0.085)	(-0.209,-0.011)	(-0.872,-0.666)	(-0.081, 0.123)	(-0.059, 0.136)
A2(+)	0.433*	-0.004	0.557*	-0.054	0.042
	(0.362, 0.528)	(-0.092, 0.065)	(0.465, 0.627)	(-0.157, 0.003)	(-0.025, 0.129)
A3(-)	0.192*	-0.577*	-0.566*	-0.067	0.140*
	(0.090, 0.299)	(-0.679,-0.465)	(-0.682,-0.471)	(-0.166, 0.048)	(0.029, 0.232)
A4(+)	0.013	-0.001	0.980*	-0.018	-0.002
	(-0.008, 0.168)	(-0.097, 0.047)	(0.892, 1.045)	(-0.093, 0.039)	(-0.062, 0.070)
A5(-)	-0.165*	-0.038	-0.815*	0.006	0.089*
	(-0.258,-0.097)	(-0.108, 0.049)	(-0.892,-0.723)	(-0.074, 0.084)	(0.012, 0.164)
A6(+)	-0.054	-0.186*	0.718*	0.011	0.013
	(-0.136, 0.042)	(-0.278,-0.104)	(0.628, 0.810)	(-0.082, 0.096)	(-0.077, 0.095)
A7(-)	-0.366*	-0.093*	-0.732*	0.070*	0.031
	(-0.458,-0.300)	(-0.169,-0.020)	(-0.798,-0.637)	(0.006, 0.157)	(-0.047, 0.099)
A8(+)	0.110*	-0.042	0.692*	0.076*	0.027
	(0.044, 0.190)	(-0.130, 0.013)	(0.618, 0.771)	(0.001, 0.147)	(-0.034, 0.107)
A9(+)	0.113*	-0.115*	0.752*	0.062	0.113*
	(0.047, 0.207)	(-0.212,-0.056)	(0.669, 0.837)	(-0.010, 0.150)	(0.041, 0.195)
A10(+)	0.432*	0.069	0.320*	0.112	0.053
	(0.348, 0.513)	(-0.007, 0.151)	(0.245, 0.402)	(-0.004, 0.158)	(-0.019, 0.138)
C1(+)	0.096	0.089	-0.039	0.682*	0.133*
	(-0.004, 0.178)	(-0.005, 0.181)	(-0.098, 0.089)	(0.563, 0.754)	(0.064, 0.246)
C2(-)	0.056	-0.180*	0.110	-0.658*	0.145*
	(-0.000, 0.206)	(-0.262,-0.050)	(-0.022, 0.181)	(-0.798,-0.578)	(0.009, 0.212)
C3(+)	-0.007	-0.007	0.112*	0.399*	0.284*
	(-0.091, 0.071)	(-0.111, 0.052)	(0.050, 0.210)	(0.302, 0.473)	(0.218, 0.382)
C4(-)	-0.107*	-0.604*	0.051	-0.478*	-0.041*
	(-0.169,-0.005)	(-0.670,-0.496)	(-0.048, 0.123)	(-0.544,-0.371)	(-0.174,-0.008)
C5(+)	0.093	0.030	-0.002	0.779*	-0.051
	(-0.020, 0.169)	(-0.091, 0.113)	(-0.048, 0.154)	(0.679, 0.881)	(-0.122, 0.072)
C6(-)	0.003	-0.172*	0.048	-0.704*	0.088
	(-0.074, 0.139)	(-0.255,-0.035)	(-0.081, 0.136)	(-0.837,-0.608)	(-0.028, 0.187)
C7(+)	-0.121*	-0.150*	0.109*	0.535*	0.040
	(-0.219,-0.041)	(-0.267,-0.085)	(0.038, 0.216)	(0.464, 0.653)	(-0.022, 0.158)
C8(-)	-0.000	-0.268*	-0.240*	-0.518*	-0.000
	(-0.073, 0.109)	(-0.340,-0.155)	(-0.355,-0.173)	(-0.604,-0.413)	(-0.123, 0.058)
C9(+)	0.053	-0.029	0.121*	0.725*	-0.076
	(-0.062, 0.125)	(-0.177, 0.024)	(0.055, 0.243)	(0.639, 0.841)	(-0.149, 0.040)
C10(+)	-0.022	-0.025	0.126*	0.523*	0.238*
	(-0.116, 0.050)	(-0.146, 0.025)	(0.068, 0.234)	(0.431, 0.609)	(0.172, 0.340)

Table K.3: Part III: Point estimates and confidence intervals constructed by L^1, big-five personality test.

	E	ES	A	C	I
I1(+)	0.003	0.002	-0.047	-0.007	0.630*
	(-0.037, 0.131)	(-0.113, 0.060)	(-0.147, 0.015)	(-0.106, 0.062)	(0.539, 0.716)
I2(-)	0.083	-0.226*	-0.086*	0.020	-0.588*
	(-0.020, 0.157)	(-0.297,-0.121)	(-0.185,-0.017)	(-0.047, 0.128)	(-0.683,-0.505)
I3(+)	0.004	-0.152*	0.023	-0.001	0.595*
	(-0.038, 0.131)	(-0.254,-0.092)	(-0.058, 0.099)	(-0.085, 0.080)	(0.501, 0.668)
I4(-)	0.105	-0.209*	-0.153*	0.046	-0.578*
	(-0.020, 0.153)	(-0.273,-0.098)	(-0.225,-0.059)	(-0.028, 0.146)	(-0.660,-0.484)
I5(+)	0.165*	0.065	-0.060	0.164*	0.586*
	(0.109, 0.252)	(-0.003, 0.138)	(-0.127, 0.006)	(0.054, 0.194)	(0.509, 0.657)
I6(-)	-0.149*	-0.004	-0.046	0.038	-0.515*
	(-0.264,-0.090)	(-0.065, 0.108)	(-0.122, 0.043)	(-0.034, 0.140)	(-0.608,-0.432)
I7(+)	0.013	0.168*	-0.036	0.087	0.455*
	(-0.044, 0.099)	(0.088, 0.229)	(-0.099, 0.037)	(-0.010, 0.135)	(0.384, 0.528)
I8(+)	-0.095	-0.164*	-0.108*	-0.001	0.664*
	(-0.177, 0.011)	(-0.276,-0.091)	(-0.194,-0.017)	(-0.097, 0.091)	(0.572, 0.768)
I9(+)	-0.081	-0.220*	0.239*	0.111*	0.262*
	(-0.149, 0.014)	(-0.321,-0.159)	(0.159, 0.318)	(0.042, 0.208)	(0.182, 0.343)
I10(+)	0.158*	-0.005	-0.002	0.086*	0.692*
	(0.110, 0.259)	(-0.114, 0.038)	(-0.068, 0.070)	(0.006, 0.158)	(0.613, 0.769)

References

Bernaards, C. A., & Jennrich, R. I. (2005). Gradient projection algorithms and software for arbitrary rotation criteria in factor analysis. *Educational and Psychological Measurement*, *65*(5), 676–696.

Finch, H. (2006). Comparison of the performance of varimax and promax rotations: Factor structure recovery for dichotomous items. *Journal of Educational Measurement*, *43*(1), 39–52.

Fitzpatrick, P. (2009). *Advanced Calculus* (Vol. 5). American Mathematical Soc.

Harman, H. H., & Harman, H. H. (1976). *Modern Factor Analysis*. University of Chicago press.

Hendrickson, A. E., & White, P. O. (1964). Promax: A quick method for rotation to oblique simple structure. *British Journal of Statistical Psychology*, *17*(1), 65–70.

Higham, N. J. (1988). Matrix nearness problems and applications. In M. Gover & S. Barnett (Eds.), *Applications of Matrix Theory* (pp. 1–27). Oxford University Press.

Jennrich, R. I. (2006). Rotation to simple loadings using component loss functions: The oblique case. *Psychometrika*, *71*(1), 173–191.

O'Rourke, S., Vu, V., & Wang, K. (2018). Random perturbation of low rank matrices: Improving classical bounds. *Linear Algebra and its Applications*, *540*, 26–59. https://doi.org/https://doi.org/10.1016/j.laa.2017.11.014

Petersen, K. B., & Pedersen, M. S. (2012). The matrix cookbook [Version 20121115]. http://www2.compute.dtu.dk/pubdb/pubs/3274-full.html

Sohrab, H. H. (2003). *Basic Real Analysis*. Springer.

Weyl, H. (1912). Das asymptotische verteilungsgesetz der eigenwerte linearer partieller differentialgleichungen (mit einer anwendung auf die theorie der hohlraumstrahlung). *Mathematische Annalen*, *71*(4), 441–479.

Yates, A. (1987). *Multivariate Exploratory Data Analysis: A Perspective on Exploratory Factor Analysis*. Suny Press.
